# Comparing Middle Latency Response With And Without Music

**DOI:** 10.1016/S1808-8694(15)30991-5

**Published:** 2015-10-19

**Authors:** Tatiane Eisencraft, Mariana Figueiredo de Miranda, Eliane Schochat

**Affiliations:** a Master's degree student at the Sao Paulo University Medical College. Program: Rehabilitation Science. Speech therapist; b Specialization in Auditory Processing; c Doctor and Professor of the Speech Therapy Course at Sao Paulo University

**Keywords:** electrophysiology, music, auditory perception

## Abstract

Auditory evoked potentials can be used as a tool to investigate the central nervous system and structures that can be activated by auditory stimulation. There are few studies correlating the Middle Latency Response with different types of auditory stimulation, which led us to undergo this study.

**Aim:**

to verify The Middle Latency Response (MLR) in normal hearing adults when stimulated by clicks and music in the contralateral ear.

**Study design:**

a cross-sectional contemporary cohort.

**Method:**

MLR was carried out on 10 normal hearing subjects using bilateral clicks (70 dB nNA) and music in the contralateral ear. We measured and compared the amplitude and latency of the Pa wave with clicks and clicks and music. We compared the amplitude and latency of the electrodes in sites C3 and C4 for both ears with and without music.

**Results:**

All subjects had MLR within normal limits for both amplitudes and latencies bilaterally. Stimuli with music and clicks revealed a reduction of the amplitude in the contralateral ear with the music stimulus in all electrode sites although this reduction was not statiscally significant.

**Conclusion:**

We conclude that music in the contralateral ear reduces the amplitude of the Pa wave of the MLR.

## INTRODUCTION

The Middle Latency Auditory Evoked Potential (MLAEP) is described as a series of waves observed in a 10 to 80 millisecond interval following an auditory stimulus^1^.

A recording of these potentials reflects cortical activity involved in primary auditory abilities (recognition, discrimination and figure-background) and non-primary auditory abilities (selective attention, auditory sequence and auditory/visual integration)^1^.

Adult cognitive function is based on specialized neural networks. In the auditory system there are different neural networks in the temporal lobe that are involved in representing different types of acoustic or sound stimulus^2^.

The middle latency response (MLR) strongly matches the behavioral auditory threshold in a given individual^3^, and can provide relevant information about the integrity of the central auditory nervous system.

The MLAEP appears to have multiple generators, with a greater participation of thalamic-cortical pathways and a lesser contribution from the inferior colliculus and the reticular formation (midbrain)^1^.

In the MLAEP it is possible to recognize four positive and three negative waves, although usually only the Pa (30ms), Pb (50ms), Na (18ms) and Nb (40ms) waves are analyzed due to their increased amplitude and stability compared to other waves^4^.

Various electrodes placed in a variety of points are required in recording this potential to assess central auditory function. A clinically feasible set up that provides a good diagnosis is to place the electrodes in positions C3, C4 and Cz (the 10-20 Electrode System of the International Federation in Electroencephalography and Clinical Neurophysiology). This placement method enables a comparison of latencies and amplitudes between each hemisphere and the midline. Inverted electrodes may be placed on the ear lobe or the mastoid of the ipsilateral or contralateral ear that is being stimulated, with a ground electrode placed on the vertex^5^.

Wave analysis is a comparative intrasubject and interhemispheric observation. Interhemispheric comparison in the same patient is more important than an intersubject comparison to establish normalcy^6^.

Auditory evoked potential responses may be used clinically to identify cortical injury and dysfunction. Other clinical applications include neuropsychiatric evaluations, such as in autism, other disorders affecting the central and peripheral auditory systems^7^, injury location, and intrasurgical monitoring.

The auditory evoked potential, however, still requires further study on sensitivity and specificity. A study by Schochat et al., (2004)^8^ at the cutoff point used in their study, found sensitivity and specificity rates of approximately 70% for subjects with central auditory nervous system injury or auditory processing disorders, and that cutoff points of 30% and 40% (the ear or the electrode effect) are those that have the best balance between sensitivity and specificity^1^.

A further clinical factor to be considered for diagnosis when using the MLR is intersubject variability, which may be seen in the Na-Pa wave amplitude^4^.

There is a need to increase the reliability and reduce the variability of this method. The auditory evoked potential response using other stimuli in place of the traditional click needs to be known. Other than the abovementioned uses, different stimuli could bring further data concerning the workings of the central auditory nervous system, as the sources generating auditory evoked potentials are located mostly in primary auditory areas.

Future promising trials could be done adding noise to the MLR assessment^9^. Studies along this line have shown that this procedure, as in certain behavioral evaluations, could increase sensitivity compared to the assessment without noise.

Considering the paucity of studies on the MLAEP with different types of auditory stimuli, our study attempted to assess MLAEP in normal-hearing adults stimulated by clicks and contralateral music, comparing Pa wave amplitude in electrodes C3 and C4 and in both ears.

## METHODS

This research protocol was submitted to the Research Ethics Committee of the Sao Paulo University Medical College Clinical Hospital (HC - FMUSP).

Inclusion criteria were: normal-hearing adults; type A tympanometry^10^, and age between 20 and 32 years. Adults were chosen due to the 20% to 90% increased possibility of detecting and recording Pa waves^11^ after puberty, as myelinization of the thalamic-cortical pathway and the sensory cortex continues until this age^1^. Ten female subjects were assessed (this number was established based on a statistical study done at the Sao Paulo University Mathematics and Statistics Institute). Previous studies using male and female subjects did not show significant differences in performance between men and women with regards to the theme of our study^12^. All participants read and signed a free and informed consent form before data collection.

Basic auditory evaluation was done initially (audiometry and imitanciometry), followed by the MLAEP electrophysiological test using clicks, in a silent environment; stimuli were presented monaurally at 9.8 clicks per second and 70 dBnHL. The number of scans was 1,000 clicks, using a 72 millisecond recording window.

Electrodes were placed over both mastoid processes (A1 and A2), on the temporal lobes or the right and left coronal regions (C3 and C4) and on the forehead (A - ground).

Scalp sites where electrodes were to be attached were cleaned to reduce electrical impedance between the skin and electrodes to less than 5 ohms.

Stimuli were emitted through headphones and responses were recorded twice for each condition (C3A1, C4A1, C3A2, C4A2) to increase reliability.

Latency measurement, done at the wave peak, was restricted to the more robust Pa wave^5^.

Recordings obtained under a common condition (ear or electrode) were compared, in other words, each tracing was compared with two other tracings. For example, the C3A1 recording was compared with C3A2 recordings (an electrode in common) and C4A1 recordings (an ear in common).

The same procedure under similar rules was done subsequently but upon a music stimulus (Ravel's Bolero and Offenbach's Orpheus in the Underworld Overture) at a comfortable intensity for the subject, in the contralateral ear to that receiving the clicks.

This procedure was done for both ears and again latency and amplitude were measured as in the first test.

Student's T test was applied to verify any significant difference between Pa wave amplitudes. The significance level was 5%.

## RESULTS

100% of subjects had middle latency responses within normal limits for both ears when using bilateral clicks at 70 dBnHL to assess the middle latency potential^6^. Results are shown on Table 1.

**Table 1:** Comparison of middle latency potential amplitude and latency values.

	C3A1		C3A2		C4A2		C4A1	
Subjects	Amplitude	Latency	Amplitude	Latency	Amplitude	Latency	Amplitude	Latency
1	0,84	35,49	0,76	34,32	0,39	32,95	0,77	33,34
2	0,55	33,54	1,66	33,34	1,08	30,61	0,59	31,39
3	1,18	34,9	1,03	35,49	1,29	34,71	1,39	36,85
4	1,31	32,95	0,61	27,10	0,83	29,25	1,01	32,95
5	0,49	33,15	0,69	32,95	0,54	30,61	0,83	32,76
6	0,52	27,88	0,88	31,39	0,57	32,17	0,46	32,17
7	0,52	33,74	1,00	34,9	0,57	33,15	0,74	33,15
8	1,32	29,05	0,86	27,49	0,94	32,37	1,12	32,37
9	1,23	37,05	0,82	29,86	0,79	32,56	1,68	34,32
10	0,76	33,74	1,08	36,66	0,92	32,17	0,40	32,95
Average	0,87	33,14	0,93	32,35	0,79	32,05	0,89	33,22

Table 2 shows results of the middle latency potential evaluation with clicks and music stimulus in the contralateral ear to the ear receiving clicks, compared with middle latency potential evaluation with clicks only.

**Table 2:** Comparison of middle latency potential amplitude and latency values with music stimulus.

Subjects	C3A1		C3 A2		C4 A2		C4 A1	
	Amplitude	Latency	Amplitude	Latency	Amplitude	Latency	Amplitude	Latency
1	1,29	37,24	1,18	32,37	0,69	32,56	1,13	32,95
2	0,75	32,37	1,56	32,95	0,60	31,00	0,98	29,83
3	0,80	31,00	1,73	36,27	1,21	31,98	1,25	32,56
4	0,53	32,95	0,68	32,76	0,73	32,17	0,91	33,74
5	0,39	31,39	0,20	33,34	0,33	30,22	0,56	31,39
6	0,60	32,95	0,40	27,10	0,46	29,05	0,49	31,98
7	0,83	32,37	0,50	33,74	0,58	34,90	0,67	34,51
8	0,62	31,78	0,73	27,30	0,97	30,61	0,63	34,51
9	0,31	26,52	0,63	34,90	0,78	35,10	0,43	33,74
10	0,89	39. 00	0,94	34,90	0,59	31,78	0,50	39,78
Average	0,70	32,30	0,85	35,83	0,69	31,93	0,75	33,49

Table 3 contains amplitude values with and without music stimulus and Student's T test values.

**Table 3:** Pa wave average, mean, standard deviation (SD) and p-value with and without a music stimulus.

	Pa amplitude - without music			Pa amplitude - with music			
	average	mean	SD	average	mean	SD	p-value
C3A2	0,94	0,87	0,29	0,85	0,7	0,49	0,52
C4A2	0,79	0,81	0,28	0,69	0,64	0,25	0,21
C3A1	0,87	0,8	0,35	0,7	0,68	0,28	0,29
C4A1	0,9	0,8	0,4	0,75	0,65	0,29	0,35

As we can see, although amplitude differences were not significant in any electrode position, all of them showed reduced amplitude with a music stimulus.

## DISCUSSION

Electrophysiological tests may significantly increase the reliability of clinical assessments, supporting and changing clinical procedures into a neurological diagnosis, as well as deepening our understanding of central auditory nervous system development and maturation^13^, ^14^.

Analysis was made using the Pa wave; according to Kraus, Kileny and McGee, 1994^1^, this is the most reliable MLAEP wave. It is also generated in auditory reception areas, namely the temporal lobe. Hall in 1992^5^ states that the Pa wave is generally the most robust middle latency wave, comparable in this sense to the V wave in brainstem auditory evoked potentials. It may be said that this potential has many generators, with a greater contribution from thalamic-cortical structures and a lesser contribution from the inferior colliculus and the reticular formation.

Some electrophysiology studies show that wave amplitude is greater in the right hemisphere, while other studies show the opposite. Such studies are not conclusive, and these results may depend on the stimulus or the required function, or may be the result of existing morphological asymmetries or the number of Sylvian fissures and hemispheric specialization. As seen on Table 1 and 2, there was no voltage difference between right and left hemisphere amplitudes in all evaluation conditions^2^.

The number of contralateral auditory pathway fibers is always higher compared to the ipsilateral pathway, which would lead us to expect that contralateral pathway amplitudes (C3A2 and C4A1) would be higher that ipsilateral amplitudes (C3A1 and C4A2). This prevalence, however, usually can only be seen in a dichotic situation, that is, when the subject is exposed to different stimuli, one for each ear; in such situations, the ipsilateral pathway is suppressed in favor of the contralateral pathway^15^. The difference could not be identified in this study, as seen on Table 2. Possibly this similarity between ipsilateral and contralateral pathway responses is due to the small sample number.

The comparison between Pa wave amplitudes with and without music stimuli in most subjects revealed reduced amplitudes when a music stimulus was presented to the contralateral ear in relation to the ear receiving clicks (see Table 3). Table 3 also shows that on average, amplitudes were higher without music stimuli in all electrode positions, although this difference was not statistically significant, possibly due to the small sample number.

The masking effect of music in the contralateral ear on Pa wave amplitude did not differ significantly between electrodes on both sides (C3 versus C4). Thus, neither hemispheric lateralization nor specialization explains the Pa wave attenuation due to the masking effect produced by music stimulus.

Although auditory evoked potentials do not allow the location of all cortical regions activated during cognitive activity^2^, different voltages (amplitudes) may be seen, suggesting different activated neural networks, as seen in this study.

Salo et al.^16^ in 2003 examined the effect of contralateral masking with white noise on cortical auditory potentials (N1 and P2) and found a significant reduction in the N1 wave amplitude when using white noise at 75 dBHL intensity, not seen in the P2 wave. They suggest that this effect may have occurred because of the efferent auditory system; we also believe that this is a probable hypothesis to explain the attenuation we found in our study. This hypothesis refers to the existence of an afferent pathway from the cochlea to the olivary complex. External ciliated cells of the contralateral cochlea (receiving noise or music in the test) are innervated by the medial efferent system^17^. Therefore, the contralateral masking effect on N1 P2 waves or the Pa wave in the MLR may be mediated by the cochlear efferent effect, as occurs with attenuation of acoustic otoemission when there is noise in the contralateral ear mediated by the efferent pathway.

Another possible hypothesis is that there is amplitude attenuation due to an inhibitory effect caused by inattention provoked by the music stimulus, although this potential is considered as exogenous and preattentional, not sensitive to cognitive and attentional operations^18^. Other studies using another exogenous potential (P50) also found reduced wave amplitudes which the authors attributed to an attention effect^19^, ^20^.

What remains to be known is whether this reduction in absolute numbers, although not statistically significant, is truly related to attention or if this inhibitory process is related to some other process.

It is difficult to discuss the findings of this study due to the small number of published papers correlating the MLR with different sound stimuli. We can see that in the MLR evaluation using music stimuli, we saw reduced Pa wave amplitudes in all electrode positions in most subjects compared to the Pa wave amplitude with no music stimuli.

## CONCLUSIONS

In our data the reduction in absolute numbers of the MLAEP Pa wave amplitude due to a music stimulus in the contralateral ear to the ear receiving clicks suggests that a music stimulus may influence the middle latency amplitude response.

Further studies on the MLR with different sound stimuli are needed to analyze with greater precision the amplitudes and latencies of resulting waves.

We suggest that other similar studies (MLAEP with noise and/or contralateral music) be undertaken with a larger sample number including not only participants with normal development, as in our study, but also those with auditory processing disorders and proven central auditory nervous system injury, to verify the sensitivity and specificity of this procedure.
